# Early time-weighted average PEEP and mortality in sepsis-associated myocardial injury requiring invasive mechanical ventilation: a retrospective cohort study with external validation

**DOI:** 10.3389/fcimb.2026.1839805

**Published:** 2026-07-30

**Authors:** Hongfei Ge, Fangchao Chen, Lili Peng, Huayi Ma, Sixing Li, Juntao Hu, Zhanhong Tang

**Affiliations:** 1 Department of Critical Care Medicine, The First Affiliated Hospital of Guangxi Medical University, Nanning, China; 2 Department of Critical Care Medicine, Liuzhou Workers’ Hospital, Liuzhou, China; 3 Department of Emergency, The First Affiliated Hospital of Guangxi Medical University, Nanning, China; 4 Department of Nursing, Liuzhou Workers’ Hospital, Liuzhou, China

**Keywords:** cardiac troponin, mechanical ventilation, mortality, myocardial injury, positive end-expiratory pressure, sepsis

## Abstract

**Background:**

Sepsis-associated myocardial injury (SAMI) defines a cardiovascularly vulnerable population in whom ventilatory strategies may affect hemodynamic stability. Accordingly, we investigated whether higher early time-weighted average (TWA) positive end-expiratory pressure (PEEP) is associated with mortality in patients with SAMI.

**Methods:**

This retrospective study used Medical Information Mart for Intensive Care IV (MIMIC-IV) and an external cohort to examine adult intensive care unit (ICU) patients with SAMI who received invasive mechanical ventilation (IMV) within 24 h of ICU admission. Associations with ICU and in-hospital mortality were estimated using generalized propensity score (GPS) weighting in covariate-adjusted logistic regression models. Nonlinearity was evaluated using restricted cubic splines. Effect modification was examined in prespecified subgroups, and robustness was assessed through sensitivity analyses.

**Results:**

The derivation cohort included 3,421 patients, with ICU and in-hospital mortality rates of 29% and 37%, respectively. In the primary GPS-weighted covariate-adjusted model, each 1 cmH_2_O increase in early TWA PEEP was associated with higher ICU mortality (adjusted odds ratio [OR], 1.14; 95% confidence interval [CI], 1.10–1.18) and in-hospital mortality (adjusted OR, 1.11; 95% CI, 1.07–1.15). Restricted cubic spline analyses suggested a generally increasing risk pattern without a clear threshold. Subgroup analyses showed no significant effect modification, and sensitivity analyses addressing exposure definition, timing, incomplete observation, and alternative covariate adjustment yielded consistent results. In the external validation cohort (n = 285), the association remained directionally consistent for ICU mortality (OR, 1.31; 95% CI, 1.08–1.59) and in-hospital mortality (OR, 1.29; 95% CI, 1.07–1.57).

**Conclusions:**

Among patients with SAMI receiving invasive mechanical ventilation, higher early TWA PEEP was consistently associated with increased ICU and in-hospital mortality in both derivation and external validation cohorts.

## Introduction

Sepsis, characterized by life-threatening organ dysfunction arising from a dysregulated host response to infection, represents a persistent global health challenge. Recent Global Burden of Disease estimates indicate that sepsis accounted for tens of millions of incident cases and approximately 11 million deaths worldwide in 2017, highlighting its substantial contribution to critical illness and mortality (Rudd et al., 2020).

The heart is particularly vulnerable to this systemic dysregulation. Cardiac troponin elevation, widely recognized as a key objective marker for injury, is highly prevalent in sepsis (reported in 47% to 67% of patients) (Bessiere et al., 2013; Frencken et al., 2018). Crucially, the prognostic implications of this injury are profound. Substantial evidence derived from large observational cohorts and meta-analyses indicates that patients with such elevation exhibit significantly higher mortality rates, with risk estimates often approximating a two-fold increase relative to those without cardiac involvement (Zheng et al., 2023).

From a structural perspective, echocardiography reveals that elevated troponin levels in sepsis-associated myocardial injury (SAMI) are strongly correlated with right ventricular (RV) abnormalities, specifically dilatation manifested as increased end-systolic volume and systolic dysfunction characterized by reduced tricuspid annular plane systolic excursion (TAPSE). In turn, these functional impairments culminate in poor clinical outcomes, serving as independent predictors of mortality (Landesberg et al., 2014; Innocenti et al., 2022).

Taken together, these findings identify SAMI as a distinct high-risk phenotype. However, management of this phenotype is particularly challenging during respiratory support, as the optimization of cardiopulmonary interactions becomes a decisive factor for survival.

Mechanical ventilation remains a fundamental intervention for septic patients with respiratory failure, where positive end-expiratory pressure (PEEP) is routinely applied to reverse alveolar collapse and optimize oxygenation (Mahmood and Pinsky, 2018). However, the application of PEEP presents a distinct hemodynamic challenge. Mechanistically, higher PEEP levels elevate intrathoracic pressure, which impedes venous return while simultaneously increasing pulmonary vascular resistance, thereby imposing increased afterload on the RV (Luecke and Pelosi, 2005; Corp et al., 2021). This concern is particularly relevant in sepsis, where RV dysfunction is not uncommon and has been associated with increased mortality risk in pooled analyses and early echocardiography cohorts (Mahmood and Pinsky, 2018; Corp et al., 2021). Under these conditions, the cardiovascular system may be less able to compensate for PEEP-induced shifts in preload and RV afterload (Mahmood and Pinsky, 2018). Consequently, PEEP strategies guided primarily by oxygenation targets could plausibly precipitate hemodynamic deterioration in patients with concomitant sepsis-related myocardial injury.

Despite this physiologic conflict, current guidelines for sepsis-related respiratory failure predominantly emphasize lung-protective strategies derived from ARDS trials (Brower et al., 2004; Evans et al., 2021), often with insufficient consideration for the specific hemodynamic fragility associated with myocardial injury. Consequently, the optimal PEEP strategy for septic patients with concurrent myocardial injury remains uncertain, particularly regarding how to balance oxygenation targets against cardiovascular burden. It is therefore clinically imperative to evaluate the association between early PEEP levels and mortality in this high-risk subgroup.

To address this clinical uncertainty, we conducted a retrospective observational cohort study utilizing data from the Medical Information Mart for Intensive Care IV (MIMIC-IV) database and an independent external validation cohort. Specifically, we investigated the association between early PEEP exposure, quantified as the time-weighted average (TWA) PEEP during the first 48 h of ventilation, and intensive care unit (ICU) and in-hospital mortality in adult patients with SAMI. By clarifying this relationship, our study aims to provide evidence to support more individualized ventilatory decision-making in this high-risk population.

## Methods

### Study design and data sources

This retrospective observational cohort study utilized data from two sources: the Medical Information Mart for Intensive Care IV (MIMIC-IV), a publicly accessible, de-identified critical care database including comprehensive data from over 50,000 patients admitted between 2008 and 2022, and an independent external validation cohort retrieved from Liuzhou Workers’ Hospital electronic medical records (including both hospital and intensive care information systems) between 1 August 2021 and 1 December 2025. The study adheres to the Strengthening the Reporting of Observational Studies in Epidemiology (STROBE) guidelines (von Elm et al., 2007). The study protocol was approved by the Ethics Committee of Liuzhou Workers’ Hospital (Approval No. KY2026612). The requirement for informed consent was waived due to the retrospective design and the use of de-identified data.

### Study population

We screened adult ICU admissions with a length of stay exceeding 24 h. In MIMIC-IV, sepsis was identified according to the Sepsis-3 definition as suspected infection with a Sequential Organ Failure Assessment (SOFA) score ≥ 2 (Singer et al., 2016). In the external cohort, sepsis was identified using International Classification of Diseases (ICD) diagnosis codes due to differences in data availability. Myocardial injury was defined as the first cardiac troponin T (cTnT) value measured within 24 h of ICU admission exceeding the assay-specific 99th percentile upper reference limit (URL). The positivity thresholds were cTnT > 0.01 ng/mL in MIMIC-IV and cTnT > 14 pg/mL in the external validation cohort. To differentiate SAMI from primary cardiac pathology or other major causes of troponin elevation, we excluded patients with: acute coronary syndrome, cardiac arrest, cardiomyopathy, valvular heart disease, myocarditis, pericarditis, endocarditis, cardiac surgery, aortic dissection, or end-stage renal disease. Patients were included if invasive mechanical ventilation (IMV) was initiated within 24 h of ICU admission. This restriction was used to align SAMI identification, baseline covariate assessment, and early PEEP exposure within the initial ICU period. For patients with multiple ICU admissions, only the first admission was analyzed.

### Data collection and management

Data extraction and curation were performed using the DecisionLinnc software. We collected patient demographics, admission characteristics, comorbidities, laboratory values, physiologic measurements (including vital signs and blood gases), and major ICU interventions. Baseline values were defined as the first measurement recorded within 24 h of ICU admission. Variables with more than 30% missingness were excluded from the analysis. Missing baseline covariates with less than 30% missingness were imputed using chained equations with 5 imputed datasets. Early TWA PEEP and outcome indicators were included only as predictors in the imputation model. Missingness of candidate variables in the derivation and external validation cohorts is summarized in **Supplementary Table S1**.

### Exposure: time-weighted average PEEP

Early PEEP exposure was defined as the time-weighted mean PEEP during the first 48 h after initiation of IMV (Algera et al., 2020; Goossen et al., 2025). This window was chosen to reflect early ventilator management after intubation, when PEEP is frequently adjusted and early airway pressure exposure may be clinically meaningful. Ventilator settings were extracted from the MIMIC-IV derived table mimiciv_derived.ventilator_setting, which includes PEEP measurements charted during ICU care. To account for irregular measurement intervals, we applied a last observation carried forward (LOCF) approach, assuming that PEEP remained constant between consecutive recorded measurements. For patients who died or were extubated within the 48-h window, the last recorded PEEP value was carried forward until the end of the 48-h period to calculate the time-weighted average.

### Covariates

Covariates for the adjusted models were selected *a priori* based on clinical knowledge and a directed acyclic graph (DAG; **Supplementary Figure S1**). Post-baseline variables were excluded to reduce bias. The prespecified covariates included age, sex, Charlson Comorbidity Index (CCI), Acute Physiology and Chronic Health Evaluation II (APACHE II) score, partial pressure of arterial oxygen (PaO_2_), fraction of inspired oxygen (FiO_2_) at IMV initiation, mean arterial pressure (MAP), heart rate (HR), respiratory rate (RR), arterial pH, partial pressure of arterial carbon dioxide (PaCO_2_), lactate, cTnT, and vasopressor therapy.

### Outcomes

The primary outcome was ICU mortality. The secondary outcome was in-hospital mortality.

### Statistical analysis

Continuous variables are presented as median and interquartile range (IQR). The Wilcoxon rank-sum test was used for between-group comparisons of continuous variables. Categorical variables are presented as counts and percentages and were compared using the chi-square test, with Fisher’s exact test used for sparse data.

Because early TWA PEEP was analyzed as a continuous exposure, generalized propensity score (GPS) weighting was used to reduce baseline differences across PEEP levels. The GPS model was constructed using the prespecified baseline covariates listed above. Stabilized weights were trimmed to limit the influence of extreme values, and balance after weighting was assessed using absolute correlations between early TWA PEEP and baseline covariates. The association between early TWA PEEP and mortality was then estimated using weighted covariate-adjusted logistic regression. Secondary analyses included unweighted univariable regression, GPS-weighted univariable regression, and unweighted multivariable regression. Results from alternative model specifications were directionally consistent, although estimates from the weighted univariable model were less precise in the smaller external cohort. Using the primary model specification, we explored nonlinear associations via restricted cubic splines with 4 knots. Odds ratios were calculated relative to 5 cmH_2_O, a value that is routinely used as a low PEEP level in mechanically ventilated patients and that approximated the lower central range of early TWA PEEP in the derivation cohort (Silva and Rocco, 2018). Curves were plotted over the 1st to 99th percentiles to ensure stability. Wald-type joint tests were used to assess nonlinearity.

To assess consistency, we applied the primary model across subgroups stratified by age (< 65 vs. ≥ 65 years), SOFA score (< 8 vs. ≥ 8), and baseline cTnT (≤ 0.11 vs. > 0.11 ng/mL). The cTnT and SOFA cutoffs were based on cohort medians to preserve subgroup size and statistical stability for interaction testing. We also assessed subgroups defined by vasopressor use and the presence of COPD. Interactions were evaluated by adding exposure by subgroup product terms.

Finally, we assessed the robustness of our findings through several sensitivity analyses. These included: (1) redefining early TWA PEEP using alternative exposure windows of 0–24 h and 0–72 h after IMV initiation; (2) performing a 48-h landmark analysis restricted to patients who remained at risk at 48 h after IMV initiation, with subsequent mortality evaluated after the landmark time point; (3) broadening the eligibility criterion for IMV initiation from within 24 h to within 48 h after ICU admission; (4) repeating the primary analysis after excluding patients who died or were discharged from the ICU before completion of the 48-h PEEP exposure window; and (5) further adjustment for SOFA score, time to IMV initiation, or ventilator mechanics, including tidal volume per kilogram and plateau pressure. All analyses were performed in R 4.5.2 or DecisionLinnc software, with two-sided P values < 0.05 considered statistically significant.

## Results

### Baseline characteristics and covariate balance

**Figure 1:**
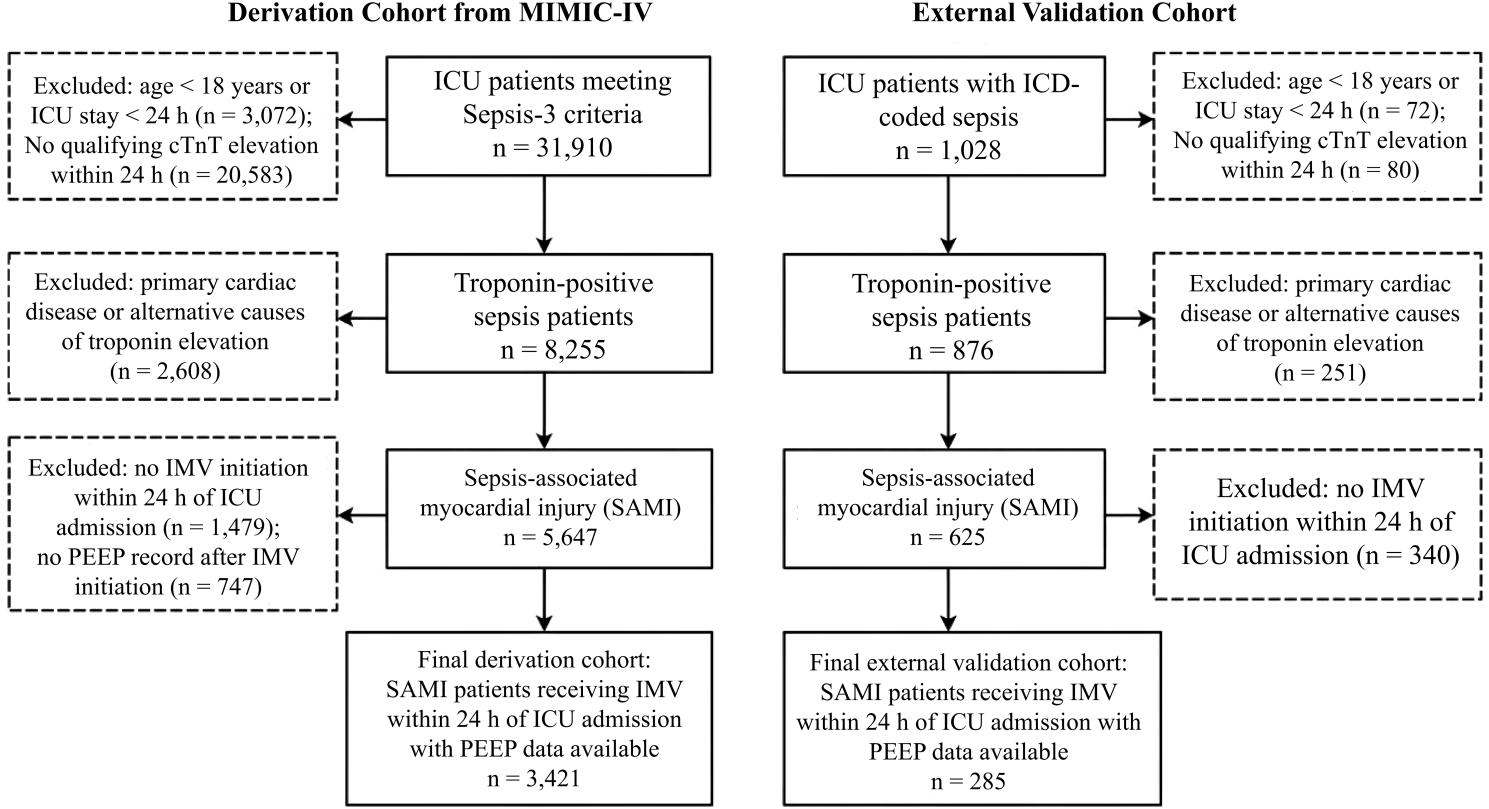
Flow chart of the study population. Troponin positivity was defined as cardiac troponin T (cTnT) > 14 pg/mL in the external validation cohort. Detailed SAMI definitions and exclusion criteria are provided in the Methods. ICU, intensive care unit; IMV, invasive mechanical ventilation; PEEP, positive end-expiratory pressure; SAMI, sepsis-associated myocardial injury.

Among the 3,421 eligible patients included in the primary cohort (**Figure 1**), the median age was 70 years (IQR 59–80) and 59% were male, with ICU and in-hospital mortality of 29% and 37%, respectively. The median early TWA PEEP was 5.5 cmH_2_O (IQR 5.0–8.4), based on a median of 13 PEEP records per patient (IQR 8–15), with a median recording interval of 210 minutes (**Supplementary Figure S2**).

Baseline characteristics were summarized according to early TWA PEEP < 8 or ≥ 8 cmH_2_O (**Table 1**). The 8 cmH_2_O cutoff was used only to characterize patients with relatively higher PEEP exposure, whereas early TWA PEEP was modeled as a continuous exposure in all primary analyses. Patients in the high-PEEP group (≥ 8 cmH_2_O) were significantly younger (65 vs. 72 years, P < 0.001) and more frequently male compared to the low-PEEP group. However, the high-PEEP group exhibited substantially greater illness severity, evidenced by higher APACHE II (26 vs. 21, P < 0.001) and SOFA scores (10.0 vs. 7.0, P < 0.001). Consistent with the requirement for increased ventilatory support, indices of respiratory mechanics and gas exchange were markedly worse in the high-PEEP group. Specifically, these patients presented with higher plateau pressures (23 vs. 17 cmH_2_O), more pronounced hypercapnia (PaCO_2_ 45 vs. 40 mmHg), and elevated lactate levels, indicating substantial baseline differences between groups.

**Table 1:** Baseline characteristics of the derivation cohort stratified by early TWA PEEP group.

Characteristic	< 8 N = 2,433	≥ 8 N = 988	P value
Demographics			
Age, years	72 (61, 82)	65 (53, 75)	< 0.001
Sex			< 0.001
Female	1,053 (43%)	347 (35%)	
Male	1,380 (57%)	641 (65%)	
Scores			
Charlson Comorbidity Index (CCI), score	6.0 (4.0, 8.0)	5.0 (3.0, 7.0)	< 0.001
APACHE II, score	21 (17, 27)	26 (20, 31)	< 0.001
SOFA, score	7.0 (5.0, 10.0)	10.0 (7.0, 12.0)	< 0.001
Glasgow Coma Scale (GCS), score	15.0 (13.0, 15.0)	15.0 (14.0, 15.0)	0.004
Ventilation & oxygenation			
Tidal volume, mL/kg	5.56 (4.63, 6.53)	5.57 (4.61, 6.61)	0.4
Plateau pressure (Pplat), cmH_2_O	17.0 (15.0, 20.0)	23.0 (20.0, 26.0)	< 0.001
FiO_2_ at IMV initiation, fraction	0.50 (0.50, 1.00)	1.00 (0.60, 1.00)	< 0.001
Vital signs			
Mean arterial pressure (MAP), mmHg	83 (71, 96)	83 (72, 95)	0.6
Heart rate (HR), bpm	89 (75, 106)	96 (82, 113)	< 0.001
Respiratory rate (RR),/min	19 (16, 24)	22 (18, 26)	< 0.001
SpO_2_, %	99.0 (96.0, 100.0)	96.0 (93.0, 99.0)	< 0.001
Body temperature, °C	36.8 (36.4, 37.2)	36.9 (36.6, 37.4)	< 0.001
Laboratory tests			
Platelets, ×10^9^/L	191 (131, 258)	190 (131, 270)	0.6
Arterial pH	7.35 (7.28, 7.41)	7.29 (7.20, 7.37)	< 0.001
PaCO_2_, mmHg	40 (35, 48)	45 (37, 56)	< 0.001
PaO_2_, mmHg	101 (53, 191)	81 (55, 122)	< 0.001
Lactate, mmol/L	1.90 (1.30, 3.10)	2.10 (1.40, 3.95)	< 0.001
White blood cell count, ×10^9^/L	13 (9, 18)	14 (9, 20)	< 0.001
Cardiac troponin T (cTnT), ng/mL	0.10 (0.04, 0.31)	0.11 (0.04, 0.33)	0.6
Creatinine, mg/dL	1.30 (0.90, 2.20)	1.60 (1.10, 2.60)	< 0.001
ALT, U/L	31 (18, 81)	45 (24, 139)	< 0.001
Prothrombin time (PT), s	15 (13, 18)	15 (13, 19)	< 0.001
Potassium, mmol/L	4.20 (3.70, 4.70)	4.40 (3.80, 5.00)	< 0.001
Hemoglobin, g/dL	10.50 (8.90, 12.30)	10.90 (9.10, 12.80)	< 0.001
Organ support			
Vasopressor use, n (%)	638 (26%)	386 (39%)	< 0.001
Renal replacement therapy (RRT), n (%)	269 (11%)	291 (29%)	< 0.001
Outcomes			
ICU mortality, n (%)	557 (23%)	424 (43%)	< 0.001
In-hospital mortality, n (%)	773 (32%)	488 (49%)	< 0.001

Values are presented as median (interquartile range) or n (%). Patients were stratified by early TWA PEEP < 8 or ≥ 8 cmH_2_O only to summarize baseline characteristics; this threshold was used to identify a higher-PEEP exposure group, whereas early TWA PEEP was analyzed as a continuous exposure in the primary models. P values are from unadjusted between-group comparisons and were not used for primary inference.

Weighting improved covariate comparability across the range of PEEP exposure. Covariate and weight diagnostics are shown in **Supplementary Figure S3**; **Supplementary Table S2**. After weighting, absolute correlations between early TWA PEEP and baseline covariates were reduced to below 0.10. After trimming weights at the 1st and 99th percentiles, the effective sample size was 2,348, corresponding to approximately 69% of the original cohort.

### Association between early TWA PEEP and mortality

In the primary adjusted weighted model, each 1 cmH_2_O increase in early TWA PEEP was associated with a 14% increase in the odds of ICU mortality (adjusted OR 1.14; 95% CI 1.10–1.18; P < 0.001) (**Table 2**). These estimates remained consistent in the conventional unweighted multivariable logistic regression (OR 1.14; 95% CI 1.11–1.17; P < 0.001).

**Table 2:** Association between early TWA PEEP and outcome in the derivation cohort.

Outcome	Model 1		Model 2		Model 3		Model 4	
	OR (95% CI)	P value	OR (95% CI)	P value	OR (95% CI)	P value	OR (95% CI)	P value
ICU mortality	1.16 (1.14–1.19)	< 0.001	1.14 (1.10–1.18)	< 0.001	1.14 (1.11–1.17)	< 0.001	1.14 (1.10–1.18)	< 0.001
In-hospital mortality	1.13 (1.10–1.15)	< 0.001	1.11 (1.07–1.14)	< 0.001	1.11 (1.08–1.15)	< 0.001	1.11 (1.07–1.15)	< 0.001

Model 1: Unweighted univariable logistic regression.

Model 2: Continuous-treatment GPS-weighted univariable model.

Model 3: Unweighted multivariable logistic regression.

Model 4: Primary model; GPS-weighted covariate-adjusted logistic regression.

Covariates (Models 3–4): age, sex, CCI, APACHE II, PaO_2_, FiO_2_ at IMV initiation, MAP, HR, RR, pH, PaCO_2_, lactate, cTnT, vasopressor use. Odds ratios are reported per 1 cmH_2_O increase in early TWA PEEP.

Similar associations were observed for in-hospital mortality. In the same primary model, each 1 cmH_2_O elevation in PEEP was associated with an adjusted OR of 1.11 (95% CI 1.07–1.15; P < 0.001). These results remained consistent in the unweighted multivariable model (OR 1.11; 95% CI 1.08–1.15; P < 0.001), confirming the stability of the association across analytical approaches.

### Restricted cubic spline analysis of early TWA PEEP and mortality

**Figure 2:**
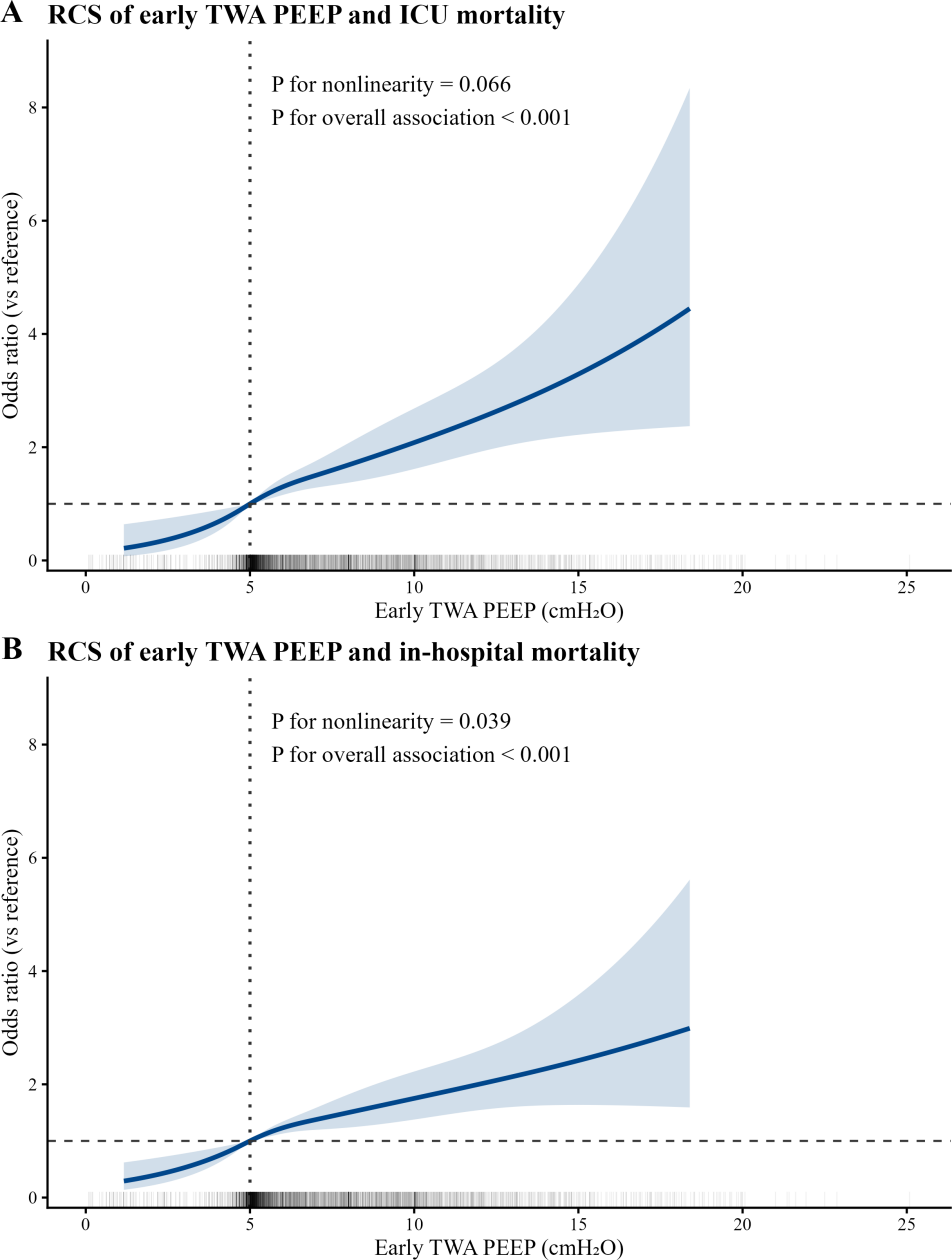
Restricted cubic spline analysis of the association between early TWA PEEP and mortality in the derivation cohort. **(A)** Association between early TWA PEEP and ICU mortality. **(B)** Association between early TWA PEEP and in-hospital mortality. Odds ratios were calculated using 5 cmH_2_O as the reference value. Shaded areas indicate 95% confidence intervals, and rug plots show the distribution of early TWA PEEP. ICU, intensive care unit; OR, odds ratio; PEEP, positive end-expiratory pressure; TWA, time-weighted average.

Restricted cubic spline analysis showed an overall increasing association between early TWA PEEP and mortality across the observed exposure range (**Figure 2**). For ICU mortality, the relationship was approximately linear (P for nonlinearity = 0.066). For in-hospital mortality, although the test for nonlinearity was statistically significant (P = 0.039), the curve remained largely monotonic and showed no clear flattening, inflection point, or plateau at the high end of PEEP exposure. The visual pattern was broadly similar to that for ICU mortality and did not suggest a distinct threshold effect. At early TWA PEEP > 15 cmH_2_O, 112 patients were observed, with 60 ICU deaths and 64 in-hospital deaths; estimates in this upper exposure range were less precise.

### Subgroup and sensitivity analyses

**Figure 3:**
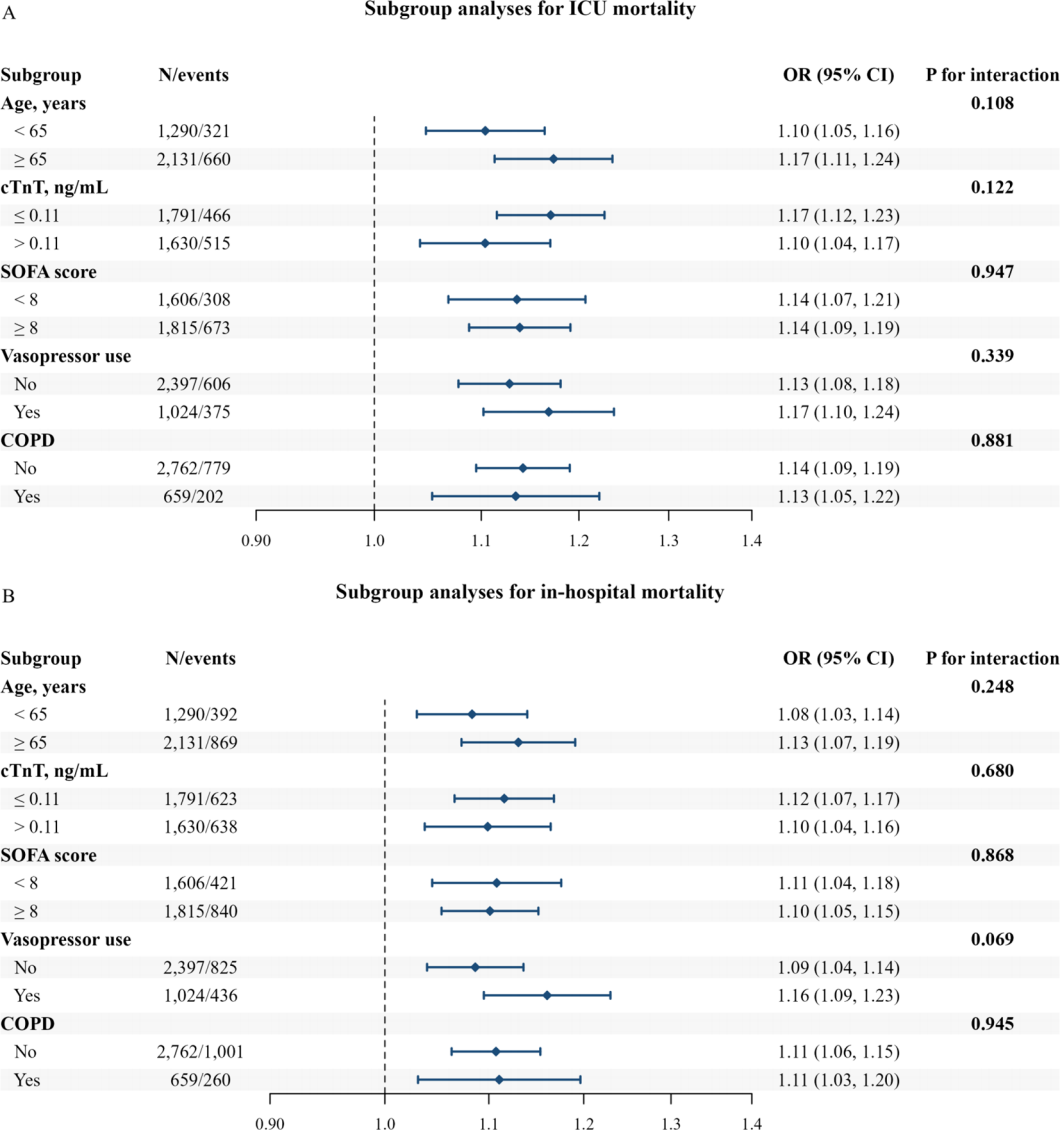
Subgroup analysis of the association between early TWA PEEP and mortality in the derivation cohort. **(A)** Subgroup analyses for ICU mortality. **(B)** Subgroup analyses for in-hospital mortality. Odds ratios are shown per 1 cmH_2_O increase in early TWA PEEP. N/events indicates the number of patients and deaths in each subgroup. The cTnT and SOFA cutoffs were based on cohort medians. CI, confidence interval; COPD, chronic obstructive pulmonary disease; cTnT, cardiac troponin T; OR, odds ratio; PEEP, positive end-expiratory pressure; SOFA, Sequential Organ Failure Assessment; TWA, time-weighted average.

Subgroup analyses demonstrated a consistent positive association between early TWA PEEP and both ICU and in-hospital mortality across all prespecified strata (**Figure 3**). There was no evidence of significant effect modification by age, baseline cTnT, SOFA score, vasopressor use, or COPD (all P for interaction > 0.05). Point estimates across all subgroups consistently exceeded 1.0 (approximately 1.08–1.17), indicating that the observed association was not driven by any single subgroup. For baseline cTnT and SOFA score, cohort medians were used as statistical stratification points for subgroup analyses rather than as established clinical thresholds; therefore, these results should be interpreted as exploratory assessments of effect modification.

**Figure 4:**
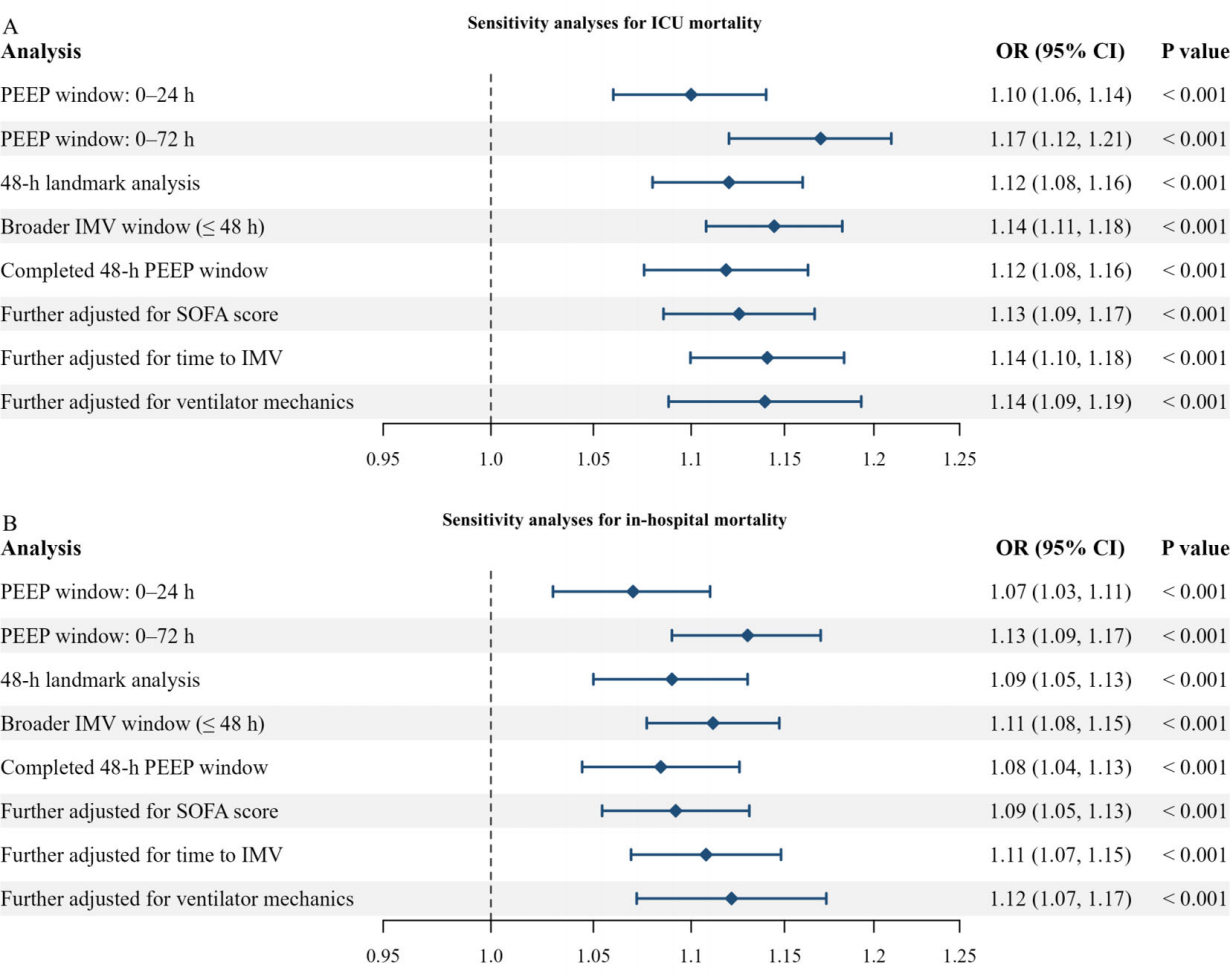
Sensitivity analyses of the association between early TWA PEEP and mortality in the derivation cohort. **(A)** Sensitivity analyses for ICU mortality. **(B)** Sensitivity analyses for in-hospital mortality. The analyses included alternative PEEP exposure windows of 0–24 h and 0–72 h after IMV initiation, a 48-h landmark analysis, broadening the IMV initiation window from within 24 h to within 48 h after ICU admission, restriction to patients who completed the 48-h PEEP exposure window, and further adjustment for SOFA score, time to IMV initiation, or ventilator mechanics, including tidal volume per kilogram and plateau pressure. CI, confidence interval; ICU, intensive care unit; IMV, invasive mechanical ventilation; OR, odds ratio; PEEP, positive end-expiratory pressure; SOFA, Sequential Organ Failure Assessment; TWA, time-weighted average.

Sensitivity analyses using the primary analytical approach further supported the robustness of the main findings (**Figure 4**). These analyses included alternative definitions of early TWA PEEP using 0–24 h and 0–72 h exposure windows, a 48-h landmark analysis, broadening of the IMV initiation window from within 24 h to within 48 h after ICU admission, restriction to patients who completed the 48-h PEEP exposure window, and further adjustment for SOFA score, time to IMV initiation, or ventilator mechanics, specifically tidal volume per kilogram and plateau pressure. For the restricted complete-observation analysis specifically addressing potential LOCF-related bias, 542 patients (15.8%) who died or whose ICU stay ended within 48 h were excluded. Across these analyses, higher early TWA PEEP remained consistently associated with increased ICU mortality, with effect estimates in the same direction and 95% CIs ranging from 1.06 to 1.21; all P values were < 0.001. Similar patterns were observed for in-hospital mortality (**Figure 4**), with directionally consistent associations and 95% CIs ranging from 1.03 to 1.17; all P values were < 0.001.

### External validation cohort

We evaluated the association in an independent external validation cohort (N = 285). In this cohort, the median early TWA PEEP was 7.20 cmH_2_O (IQR 5.41–8.73). The overall median age was 64 years (IQR 53–73), and 72% (n = 205) were male. The ICU and in-hospital mortality rates for the entire external cohort were 44% and 46%, respectively. To further characterize the clinical phenotypes associated with different PEEP levels, we stratified the cohort by a threshold of 8 cmH_2_O. This stratification revealed that patients in the higher PEEP group had significantly lower oxygenation (P < 0.001) and mean arterial pressure (P = 0.016), whereas APACHE II scores were not statistically different between groups (**Table 3**). In unadjusted analyses, higher PEEP showed a trend toward increased mortality (ICU: 53% vs. 40%, P = 0.057; in-hospital: 54% vs. 43%, P = 0.10).

**Table 3:** Baseline characteristics of the external validation cohort stratified by early TWA PEEP groups (< 8 vs. ≥ 8 cmH_2_O).

Characteristic	< 8 N = 206	≥ 8 N = 79	P value
Demographics			
Age, years	64 (52, 73)	67 (54, 73)	0.5
Sex			0.6
Female	60 (29%)	20 (25%)	
Male	146 (71%)	59 (75%)	
Scores			
APACHE II, score	26 (21, 33)	26 (20, 32)	0.4
Comorbidity index, score	1.00 (0.00, 2.00)	1.00 (0.00, 2.00)	0.8
Respiratory variables			
PaO_2_/FiO_2_	246 (198, 318)	193 (154, 249)	< 0.001
PaO_2_, mmHg	114 (95, 141)	105 (92, 130)	0.057
FiO_2_ at IMV initiation, fraction	0.50 (0.50, 0.60)	0.50 (0.50, 0.60)	0.029
Respiratory rate (RR),/min	20.0 (17.0, 24.0)	20.0 (16.0, 25.0)	0.8
SpO_2_, %	95.1 (92.5, 97.4)	95.3 (91.1, 97.4)	0.8
Vital signs			
Mean arterial pressure (MAP), mmHg	73 (66, 83)	70 (63, 77)	0.016
Heart rate (HR), bpm	93 (80, 105)	90 (77, 109)	0.3
Body temperature, °C	36.8 (36.4, 37.3)	36.8 (36.4, 37.2)	0.7
Laboratory tests			
Platelets, ×10^9^/L	123 (74, 188)	111 (80, 158)	0.2
Arterial pH	7.39 (7.35, 7.43)	7.38 (7.33, 7.42)	0.3
PaCO_2_, mmHg	39 (35, 43)	41 (35, 45)	0.1
Lactate, mmol/L	2.70 (2.00, 4.60)	3.00 (2.10, 4.90)	0.4
White blood cell count, ×10^9^/L	11.4 (8.6, 13.9)	10.3 (7.7, 15.9)	0.8
Creatinine, µmol/L	160 (91, 299)	167 (101, 259)	0.6
ALT, U/L	43 (22, 94)	36 (18, 90)	0.2
AST, U/L	77 (40, 176)	71 (41, 244)	0.7
Total bilirubin, µmol/L	21 (11, 34)	24 (14, 53)	0.051
Prothrombin time (PT), s	14.9 (13.4, 17.7)	15.0 (13.8, 16.6)	0.8
Activated partial thromboplastin time, s	46 (38, 58)	48 (39, 57)	0.9
Sodium, mmol/L	144 (139, 149)	145 (141, 149)	0.5
Potassium, mmol/L	4.00 (3.80, 4.30)	4.00 (3.70, 4.40)	0.9
Hemoglobin, g/L	80 (73, 92)	82 (70, 94)	> 0.9
Albumin, g/L	28.5 (26.4, 30.7)	28.4 (26.5, 30.4)	0.8
Glucose, mmol/L	8.45 (7.10, 11.00)	8.70 (6.90, 10.70)	> 0.9
Cardiac troponin T (cTnT), pg/mL	105 (57, 275)	84 (40, 318)	0.14
CK-MB, U/L	23 (15, 48)	24 (15, 50)	> 0.9
C-reactive protein (CRP), mg/L	113 (83, 149)	127 (88, 152)	0.2
Treatments			
Vasopressor use, n (%)	99 (48%)	34 (43%)	0.5
Renal replacement therapy (RRT), n (%)	28 (14%)	8 (10%)	0.6
Comorbidities			
Hypertension, n (%)	75 (36%)	31 (39%)	0.8
Chronic obstructive pulmonary disease, n (%)	7 (3.4%)	4 (5.1%)	0.5
Type 2 diabetes, n (%)	50 (24%)	19 (24%)	> 0.9
Hyperlipidemia, n (%)	11 (5.3%)	4 (5.1%)	> 0.9
Cerebrovascular disease, n (%)	58 (28%)	19 (24%)	0.6
Liver cirrhosis, n (%)	4 (1.9%)	3 (3.8%)	0.4
Chronic kidney disease, n (%)	18 (8.7%)	6 (7.6%)	> 0.9
Outcomes			
ICU mortality, n (%)	82 (40%)	42 (53%)	0.057
In-hospital mortality, n (%)	88 (43%)	43 (54%)	0.10

Values are presented as median (interquartile range) or n (%). Patients were stratified by early TWA PEEP < 8 or ≥ 8 cmH_2_O only to summarize baseline characteristics; this threshold was used to identify a higher-PEEP exposure group, whereas early TWA PEEP was analyzed as a continuous exposure in the primary models. P values are from unadjusted between-group comparisons and were not used for primary inference.

In the primary adjusted weighted model, each 1 cmH_2_O increase in early TWA PEEP was associated with higher odds of both ICU mortality (OR 1.31; 95% CI 1.08–1.59; P = 0.006) and in-hospital mortality (OR 1.29; 95% CI 1.07–1.57; P = 0.009). Weight diagnostics are shown in **Supplementary Figure S4**; **Supplementary Table S3**, and model estimates are shown in **Supplementary Table S4**. The positive direction of association was also observed in the unweighted and multivariable analyses shown in **Supplementary Table S4**, but estimates were less precise in the smaller external cohort, particularly after GPS weighting.

### Robustness of findings in the external cohort

Given the high completeness of PaO_2_/FiO_2_ data in the external cohort, we performed a sensitivity analysis substituting the separate PaO_2_ and FiO_2_ at IMV initiation covariates with the PaO_2_/FiO_2_ ratio (**Supplementary Table S5**). Unweighted models confirmed a significant positive association between early TWA PEEP and mortality (ICU: OR 1.16, 95% CI 1.04–1.29, P = 0.009).

After applying the same weighting approach, confidence intervals widened, but the direction and magnitude of association remained consistent. In the fully adjusted model, higher early TWA PEEP remained associated with both ICU mortality (OR 1.28, 95% CI 1.02–1.62; P = 0.037) and in-hospital mortality (OR 1.28, 95% CI 1.01–1.61; P = 0.038).

## Discussion

In this retrospective cohort study of patients with SAMI requiring early invasive mechanical ventilation, higher early TWA PEEP was associated with increased ICU and in-hospital mortality in both the MIMIC-IV derivation cohort and the external validation cohort. Overall, early TWA PEEP provided clinically relevant prognostic information during the initial ICU period. The spline analyses showed a generally increasing risk pattern across the observed PEEP range, but did not identify a clear threshold at which risk abruptly changed. For in-hospital mortality, the statistically significant nonlinearity should be interpreted in relation to the purpose of the spline test. This test examines whether the association departs from a simple straight-line pattern across the PEEP range; therefore, the significant result indicates that the association was not fully captured by a constant linear slope across PEEP levels. However, the curve remained predominantly monotonic, without a clear flattening, inflection point, or abrupt acceleration in risk. Thus, the observed nonlinearity is better described as modest curvature within an otherwise increasing association, rather than evidence of a clinically meaningful threshold. Because ICU deaths are included within in-hospital deaths, whereas in-hospital mortality additionally captures deaths occurring after ICU discharge, the two endpoints are clinically related but not identical. The broadly similar spline patterns therefore support a consistent clinical interpretation across ICU mortality and in-hospital mortality.

Higher early TWA PEEP also appeared to reflect underlying cardiopulmonary burden. In the derivation cohort, patients receiving higher PEEP were younger but had greater illness severity, worse oxygenation, greater ventilatory support needs, and higher mortality. In the external cohort, higher PEEP was likewise associated with worse oxygenation, whereas APACHE II scores were not statistically different between PEEP strata. Given the smaller sample size, high overall mortality, and descriptive nature of the PEEP stratification in the external cohort, the absence of a statistically significant APACHE II difference should be interpreted with caution. APACHE II provides a global measure of illness severity but may not fully capture differences in oxygenation impairment, hemodynamic status, ventilatory burden, or cardiopulmonary reserve. Early TWA PEEP may therefore represent both the intensity of ventilatory management and the severity of cardiopulmonary compromise, which may partly explain its consistent association with mortality across cohorts and analytical approaches.

The existing evidence guiding PEEP practice has mainly focused on ARDS. In landmark acute respiratory distress syndrome (ARDS) trials and pooled analyses, higher PEEP strategies often improved oxygenation but did not consistently improve survival, and more aggressive recruitment or high-pressure strategies have been associated with hemodynamic instability and lung injury in some settings (Meade et al., 2008; Mercat et al., 2008; Briel et al., 2010; Cavalcanti et al., 2017). Similar findings have been reported in patients without ARDS, in whom higher PEEP reduced hypoxemia without a clear mortality benefit (Pettenuzzo et al., 2021). These data suggest that the clinical effect of PEEP depends not only on oxygenation, but also on lung recruitability, overdistension, and systemic tolerance (Sousa et al., 2025; Menga et al., 2026). This uncertainty is particularly relevant in SAMI, because troponin elevation and right ventricular dysfunction are common in sepsis and are associated with adverse outcomes (Vallabhajosyula et al., 2021; Zhang et al., 2021; Shvilkina and Shapiro, 2023; Zheng et al., 2023). However, major PEEP trials did not stratify patients according to myocardial injury, right ventricular function, or cardiovascular reserve. Our study addresses this gap by evaluating early PEEP exposure in patients with SAMI, a subgroup with impaired cardiopulmonary reserve.

A plausible physiologic explanation is that PEEP affects both the lung and the circulation. PEEP can improve oxygenation by reopening collapsed alveoli and preventing end-expiratory alveolar collapse, but excessive PEEP may cause overdistension, increase pulmonary vascular resistance, and raise right ventricular load (Corp et al., 2021; Sousa et al., 2025; Menga et al., 2026). Higher PEEP also increases intrathoracic pressure, which may reduce venous return and cardiac preload in volume-dependent patients, while in some patients it may reduce left ventricular transmural pressure and lower left ventricular afterload (Mahmood and Pinsky, 2018; Corp et al., 2021; Lai et al., 2023). Therefore, the overall hemodynamic effect of PEEP depends on lung recruitability, overdistension, right and left ventricular function, and volume status. In patients with SAMI, myocardial injury and impaired cardiopulmonary reserve may narrow this balance, so higher early TWA PEEP may reflect the balance between pulmonary benefit and hemodynamic tolerance.

Clinically, our findings support a more individualized approach to PEEP titration in patients with SAMI. In these patients, a higher PEEP level should not be judged only by whether oxygenation improves. Its effects on respiratory mechanics, blood pressure, vasopressor requirement, tissue perfusion, volume status, and ventricular function should also be considered when feasible. Improved oxygenation after increasing PEEP may not necessarily indicate a favorable overall response if it is accompanied by hemodynamic deterioration. Therefore, early PEEP adjustment in SAMI should balance potential pulmonary benefit with cardiovascular tolerance.

This study has several limitations. First, because of the observational design, residual confounding and confounding by indication remain possible. Patients receiving higher PEEP may have had more severe hypoxemia, poorer lung recruitability, or greater cardiopulmonary instability, which could not be fully captured in the available data. Second, PEEP is a time-varying intervention that may be adjusted according to oxygenation, hemodynamics, sedation, fluid status, and ventilatory mechanics. In our cohort, PEEP exposure was derived from intermittent and clinically driven charting, with a median of 13 records per patient and a median recording interval of 210 minutes. This documentation pattern does not provide a continuously measured exposure trajectory and may not capture all short-term PEEP changes. Therefore, TWA PEEP estimates based on LOCF should be interpreted as an approximation of early cumulative PEEP exposure rather than a direct measurement of the true time course of PEEP. In addition, the LOCF approach used to calculate 48-h TWA PEEP in patients with incomplete observation may have introduced exposure misclassification, although results were consistent after restricting the analysis to patients who completed the 48-h PEEP exposure window. Third, several important physiologic variables were not uniformly available, including comprehensive echocardiographic data, respiratory system compliance, recruitment maneuvers, fluid balance, and dynamic measures of volume responsiveness. Fourth, troponin elevation in sepsis may arise from heterogeneous mechanisms and may not represent a uniform myocardial phenotype. Subgroup analyses were exploratory; median-based cutoffs for cTnT and SOFA simplified continuous variables, and some strata, such as COPD, included relatively few patients and events, limiting the ability to detect effect modification (Altman and Royston, 2006). Finally, the external validation cohort was smaller and identified sepsis using ICD codes, which may have reduced estimate precision and limited direct comparison with the MIMIC-IV cohort (Donnelly et al., 2020; Schwarzkopf et al., 2024). In this cohort, model estimates were directionally consistent but less stable across specifications. For ICU mortality, the weighted univariable model did not reach statistical significance (OR 1.19; 95% CI 0.96–1.47; P = 0.11), whereas the primary weighted covariate-adjusted model showed a significant positive association (OR 1.31; 95% CI 1.08–1.59; P = 0.006). This discrepancy likely reflects the smaller sample size, residual covariate imbalance, and greater sensitivity of weighted estimates to model specification. Therefore, the external validation findings should be interpreted primarily as supportive evidence for directional consistency rather than as precise replication of effect size. Prospective studies incorporating oxygenation, respiratory mechanics, hemodynamics, and cardiac function are needed to determine whether individualized PEEP adjustment can improve outcomes in patients with SAMI.

## Conclusion

Among adults with SAMI receiving IMV, higher early TWA PEEP exposure was consistently associated with increased ICU and in-hospital mortality in both derivation and external validation cohorts. These findings underscore the necessity for prospective, physiology-guided trials to define optimal PEEP adjustment strategies that carefully balance oxygenation objectives against hemodynamic tolerance in this high-risk patient population.

## Data Availability

The original contributions presented in the study are included in the article/**Supplementary Material**, further inquiries can be directed to the corresponding author/s.
