# Supplementary material for: Early time-weighted average PEEP and mortality in sepsis-associated myocardial injury requiring invasive mechanical ventilation: a retrospective cohort study with external validation
Source: Front Cell Infect Microbiol. 2026 Jul 30;16:1839805. doi: 10.3389/fcimb.2026.1839805 (PMC13468925; doi:10.3389/fcimb.2026.1839805)
Supplement: Supplementary file 1 [file DataSheet1.docx]

**Supplementary Materials**

**Supplementary Figures**

## Supplementary Figure S1. Directed acyclic graph (DAG) used to identify covariates.

**Supplementary Figure S2. Quality-control plots for early TWA-PEEP (0–48 h) in the derivation cohort.**
(A) Distribution of TWA-PEEP (cmH₂O; histogram with kernel density).
(B) Median interval between PEEP recordings in the first 48 h (min).
(C) Number of valid PEEP records contributing to TWA-PEEP (n).

(D) Violin and jitter plot showing the distribution of patient-level early TWA PEEP values.

**Supplementary Figure S3. Covariate balance in the derivation cohort.**
Absolute correlations between early TWA-PEEP and covariates are shown before weighting and after continuous-treatment GPS weighting (untrimmed and 1st/99th-percentile trimmed). The dashed line marks |r| = 0.10.

**Supplementary Figure S4. Covariate balance in the external validation cohort.**
Absolute correlations between early TWA-PEEP and covariates are shown before weighting and after continuous-treatment GPS weighting (untrimmed and 1st/99th-percentile trimmed). The dashed line marks |r| = 0.10.

**Supplementary Tables**

**Supplementary Table S1.** Variable missingness in the derivation and external validation cohorts

| **Variable** | **MIMIC-IV cohort missing,**  **n (%) (N = 3,421)** | **External validation cohort missing, n (%) (N = 285)** |
| --- | --- | --- |
| Age | 0 (0.00%) | 0 (0.00%) |
| Sex | 0 (0.00%) | 0 (0.00%) |
| Race | 0 (0.00%) | 0 (0.00%) |
| Charlson Comorbidity Index | 36 (1.05%) | 0 (0.00%) |
| APACHE II | 36 (1.05%) | 17 (5.96%) |
| SOFA | 36 (1.05%) | - |
| GCS | 36 (1.05%) | - |
| Heart rate | 0 (0.00%) | 0 (0.00%) |
| Mean arterial pressure | 55 (1.61%) | 0 (0.00%) |
| Respiratory rate | 0 (0.00%) | 0 (0.00%) |
| SpO₂ | 0 (0.00%) | 0 (0.00%) |
| Body temperature | 89 (2.60%) | 0 (0.00%) |
| PaO₂ | 63 (1.84%) | 0 (0.00%) |
| FiO₂ at IMV initiation | 5 (0.15%) | 0 (0.00%) |
| PaCO₂ | 66 (1.93%) | 0 (0.00%) |
| Arterial pH | 52 (1.52%) | 0 (0.00%) |
| PaO₂/FiO₂ ratio | 1,607 (46.97%) | 8 (2.81%) |
| Tidal volume | 33 (0.96%) | 0 (0.00%) |
| Plateau pressure | 454 (13.27%) | 0 (0.00%) |
| Early TWA PEEP, 0–48 h | 0 (0.00%) | 0 (0.00%) |
| Time to IMV initiation | 0 (0.00%) | 0 (0.00%) |
| Platelets | 19 (0.56%) | 0 (0.00%) |
| WBC | 16 (0.47%) | 0 (0.00%) |
| Hemoglobin | 13 (0.38%) | 0 (0.00%) |
| Creatinine | 1 (0.03%) | 0 (0.00%) |
| ALT | 408 (11.93%) | 0 (0.00%) |
| AST | 384 (11.22%) | 0 (0.00%) |
| Total bilirubin | 385 (11.25%) | 0 (0.00%) |
| PT | 92 (2.69%) | 0 (0.00%) |
| APTT | 107 (3.13%) | 0 (0.00%) |
| Sodium | 1 (0.03%) | 0 (0.00%) |
| Potassium | 4 (0.12%) | 0 (0.00%) |
| Glucose | 0 (0.00%) | 0 (0.00%) |
| Lactate | 134 (3.92%) | 0 (0.00%) |
| cTnT | 0 (0.00%) | 0 (0.00%) |
| CK-MB | 2,090 (61.09%) | 0 (0.00%) |
| CRP | 3,007 (87.90%) | 0 (0.00%) |
| Albumin | 1,865 (54.52%) | 0 (0.00%) |
| Vasopressor use | 0 (0.00%) | 0 (0.00%) |
| Renal replacement therapy | 0 (0.00%) | 0 (0.00%) |
| COPD | 41 (1.20%) | 0 (0.00%) |
| Hypertension | 41 (1.20%) | 0 (0.00%) |
| Type 2 diabetes | 41 (1.20%) | 0 (0.00%) |
| Hyperlipidemia | 41 (1.20%) | 0 (0.00%) |
| Cerebrovascular disease | 41 (1.20%) | 0 (0.00%) |
| Liver cirrhosis | 41 (1.20%) | 0 (0.00%) |
| ICU mortality | 0 (0.00%) | 0 (0.00%) |
| In-hospital mortality | 0 (0.00%) | 0 (0.00%) |

Dash indicates that the variable was not available or not used in that cohort.

**Supplementary Table S2.** Continuous-treatment GPS weight summary in the derivation cohort.

| **model** | **mean** | **var** | **sd** | **cv** | **min** | **p01** | **p05** | **p50** | **p95** | **p99** | **max** | **ESS** |
| --- | --- | --- | --- | --- | --- | --- | --- | --- | --- | --- | --- | --- |
| raw | 1.21 | 1.99 | 1.41 | 1.16 | 0.00 | 0.11 | 0.46 | 0.97 | 2.30 | 5.99 | 34.47 | 1453.78 |
| trim1_99 | 1.00 | 0.46 | 0.68 | 0.68 | 0.09 | 0.09 | 0.40 | 0.84 | 1.99 | 5.19 | 5.20 | 2347.99 |
| trim2_98 | 1.00 | 0.30 | 0.55 | 0.55 | 0.21 | 0.21 | 0.41 | 0.86 | 2.04 | 3.47 | 3.47 | 2622.24 |

ESS = effective sample size.

**Supplementary Table S3.** Continuous-treatment GPS weights in the external validation cohort.

| **model** | **mean** | **var** | **sd** | **cv** | **min** | **p01** | **p50** | **p99** | **max** | **ESS** |
| --- | --- | --- | --- | --- | --- | --- | --- | --- | --- | --- |
| raw | 1.53 | 11.89 | 3.45 | 2.26 | 0.02 | 0.05 | 1.00 | 14.06 | 51.19 | 46.83 |
| trim1_99 | 1.00 | 1.57 | 1.25 | 1.25 | 0.04 | 0.04 | 0.73 | 9.98 | 10.27 | 111.31 |
| trim2_98 | 1.00 | 0.54 | 0.73 | 0.73 | 0.09 | 0.09 | 0.81 | 3.99 | 3.99 | 185.47 |

ESS = effective sample size.

**Supplementary Table S4.** Association between early TWA-PEEP and outcome in the external validation cohort

| **Outcome** | **Model 1** | | **Model 2** | | **Model 3** | | **Model 4** | |
| --- | --- | --- | --- | --- | --- | --- | --- | --- |
|  | **OR**  **(95% CI)** | **P value** | **OR**  **(95% CI)** | **P value** | **OR**  **(95% CI)** | **P-value** | **OR**  **(95% CI)** | **P value** |
| ICU mortality | 1.16 (1.04–1.29) | 0.009 | 1.19 (0.96–1.47) | 0.11 | 1.31 (1.11–1.55) | 0.001 | 1.31 (1.08–1.59) | 0.006 |
| In-hospital mortality | 1.13 (1.02–1.26) | 0.026 | 1.18 (0.95–1.46) | 0.14 | 1.31 (1.11–1.55) | 0.001 | 1.29 (1.07–1.57) | 0.009 |

Model 1: Unweighted univariable logistic regression

Model 2: Continuous-treatment GPS-weighted univariable model

Model 3: Unweighted multivariable logistic regression

Model 4: primary model; GPS-weighted covariate-adjusted logistic regression

Covariates (Models 3–4): age, sex, CCI, APACHE II, PaO₂, FiO₂ at IMV initiation, MAP, HR, RR, pH, PaCO₂, lactate, cTnT, vasopressor use.

**Supplementary Table S5.** Sensitivity analysis in the external validation cohort

| **Outcome** | **Model 1** | | **Model 2** | | **Model 3** | | **Model 4** | |
| --- | --- | --- | --- | --- | --- | --- | --- | --- |
|  | **OR**  **(95% CI)** | **P-value** | **OR**  **(95% CI)** | **P-value** | **OR**  **(95% CI)** | **P-value** | **OR**  **(95% CI)** | **P-value** |
| ICU mortality | 1.16 (1.04–1.29) | 0.009 | 1.22 (0.96–1.56) | 0.10 | 1.19 (1.02–1.41) | 0.031 | 1.28 (1.02–1.62) | 0.037 |
| In-hospital mortality | 1.13 (1.02–1.26) | 0.026 | 1.21 (0.95–1.54) | 0.11 | 1.21 (1.03–1.42) | 0.022 | 1.28 (1.01–1.61) | 0.038 |

Model 1: Unweighted univariable logistic regression

Model 2: Continuous-treatment GPS-weighted univariable model

Model 3: Unweighted multivariable logistic regression

Model 4: primary model; GPS-weighted covariate-adjusted logistic regression

Covariates (Models 3–4): age, sex, CCI, APACHE II, PaO_2_/FiO_2_ ratio, MAP, HR, RR, pH, PaCO₂, lactate, cTnT, vasopressor use.
